# Patients in Iowa Counties Lacking Hospitals With Labor and Delivery Services Disproportionately Receive Care at Level III Maternal Care Hospitals When Undergoing Cesarean Delivery: A Retrospective Longitudinal Study

**DOI:** 10.7759/cureus.30683

**Published:** 2022-10-25

**Authors:** Kokila N Thenuwara, Franklin Dexter, Johannes Ledolter, Stephanie M Radke, Richard H Epstein

**Affiliations:** 1 Anesthesia, University of Iowa, Iowa City, USA; 2 Business Analytics, University of Iowa, Iowa City, USA; 3 Obstetrics and Gynecology, University of Iowa, Iowa City, USA; 4 Anesthesiology, University of Miami Miller School of Medicine, Miami, USA

**Keywords:** hospital engineering, industrial engineering, cesarean delivery, rural healthcare, obstetric anesthesia, managerial epidemiology, administrative data

## Abstract

Introduction

Many obstetrical patients from rural areas in the United States lack hospitals that provide labor and delivery care. Our objective was to examine the effects of such patients on caseloads of cesarean deliveries at Iowa hospitals with level III maternal care, as defined by the Iowa Department of Public Health (e.g., with obstetric anesthesiologists).

Methods

This retrospective longitudinal study included every discharge with cesarean delivery in the state of Iowa from October 2015 through June 2021. There were N=60,534 such deliveries from 76 hospitals, of which three were level III, and the rest were level I or II. Poisson regression models with robust variance estimation and controlling for geography, maternal risk factors, and insurance, were used to evaluate the binary outcome of whether patients received care at the university level III hospital in Eastern Iowa, or not. Similar models were also developed for care at the two private level III hospitals in Central Iowa, or not. Differences in the mean probabilities of receiving care at the level III hospitals were then estimated using logistic regression, with results reported in units of changes in cases per week at the hospitals.

Results

Statewide, the university level III hospital performed 7.4% of the cesarean deliveries, and the two private level III hospitals performed 23.4%. Patients from counties in which no cesarean deliveries were performed during the quarter of the year when they underwent a cesarean delivery disproportionately received care at level III hospitals versus levels I and II hospitals. Lower 99% confidence limits for incremental risk ratios were 1.46 and 4.20, respectively. Cesarean deliveries among patients residing in counties where no hospital had a labor and delivery ward were distributed unequally between the counties of the hospitals with level III maternal care. There were approximately 1.09 (standard error 0.10) extra cesarean deliveries per week at the university hospital versus 5.81 (standard error 0.11) at the private hospitals. The 1.09 vs 5.81 difference was caused, in part, by the effects of insurance and other hospitals with similar services.

Conclusions

Patients residing in counties without labor and delivery care disproportionately go to level III hospitals. These results can help anesthesiologists, obstetricians, and analysts at hospitals with large tertiary (level III) programs interpret their annual increases in total obstetric anesthesia activity.

## Introduction

There has been progressive closure of rural obstetric units in the United States, including those in Iowa [1-3], principally because of an inadequate obstetric workforce [4]. Statewide programs in Iowa are in various stages of implementation to alleviate the decline in patient access to cesarean delivery care (e.g., fellowships for family medicine physicians in obstetrics, with a focus on surgical care). Our previous study showed that the anesthesia workforce does not limit cesarean deliveries in Iowa [5]. Many obstetrical patients reside in counties without hospitals that provide labor and delivery care [3-5]. Our broad objective was to understand the effects of the statewide programs and patients on caseloads of cesarean deliveries at hospitals with level III maternal care [6,7]. This work is important because the previously developed and validated technique to forecast cesarean deliveries and obstetrical anesthesia workload at these hospitals uses time series analysis of data from each hospital, individually [8]. Here, we expanded considerations related to increases in cesarean deliveries at level III hospitals to include an analysis of the statewide data rather than data only from the individual hospitals.

We formulated two hypotheses. Hypothesis #1 was that patients residing in counties without hospitals offering labor and delivery services would have a proportionately greater probability of receiving care at level III hospitals, after adjusting for other covariates (e.g., maternal risk factors). In other words, if level I hospitals cease providing obstetric care, there would be more cesarean deliveries at the level III hospitals of the two counties in Iowa with such hospitals (i.e., both relative risks >1). Our rationale was that if patients cannot receive care locally, they often would go to well-known (large) hospitals [9,10]. However, that conceptual model may be inaccurate because patients may principally receive care at closer hospitals (e.g., level I with nurse anesthetists) rather than level III hospitals (e.g., with obstetric anesthesiologists) [11].

Hypothesis #2 was that the division of extra cases between level III hospitals (e.g., with anesthesia training programs versus without) would be relatively equal between the two counties with such hospitals. However, earlier studies of total counts of surgical cases between the hospitals’ counties found that geography alone was insufficient for the prediction of the distribution of cases [12]. There also were effects of insurance and other hospitals in the healthcare districts [12]. Consequently, our test of hypothesis #2, a similar distribution of the patients leaving their county for cesarean delivery between the level III hospitals’ counties, would provide insight into the potential impact on those hospitals of initiatives to abate the need for gravid women to travel substantial distances for their obstetric care.

The tests of the hypotheses are important because the results have a straightforward interpretation for the level III hospitals related to projected increases in caseload from the continuing closure of rural hospitals. Counts of cesarean deliveries show changes in total obstetric anesthesia workload because the fraction of the anesthesia workload due to cesarean deliveries versus labor analgesia was unchanging over decades [8]. Specifically, the time series analyses for the obstetric anesthesia workload of these busy hospitals use annual counts of cesarean deliveries (or cesarean deliveries plus a fraction of the annual count of labor epidurals) in linear regressions [8]. The results of hypotheses #1 and #2 provide an interpretation of the slope of the regression lines.

## Materials and methods

Ethics

The University of Iowa Institutional Review Board determined that this project (#2021112399) does not meet the regulatory definition of human subjects research because the analyses use only deidentified statewide hospital discharge data, and thus is exempt from review.

Characteristics of the studied state of Iowa

The Iowa Department of Public Health designates that Level I hospitals have facilities and personnel for cesarean delivery, blood transfusion, clinical pathology, and neonatal resuscitation [6]. All hospitals in Iowa providing labor and delivery care must have the ability to perform cesarean delivery [6]. Level II hospitals must have obstetrician(s) and pediatrician(s) on staff [6]. Level II regional centers have a further requirement for a neonatal intensive care unit [6]. Anesthesia practitioners have special training or experience in obstetric and pediatric anesthesia [6]. Level III centers must have maternal-fetal medicine obstetrician(s) and pediatric surgeon(s) [6]. Level III hospitals are mostly located in metropolitan areas [1]. These definitions and criteria are those required of hospitals in the state of Iowa.

**Figure 1:**
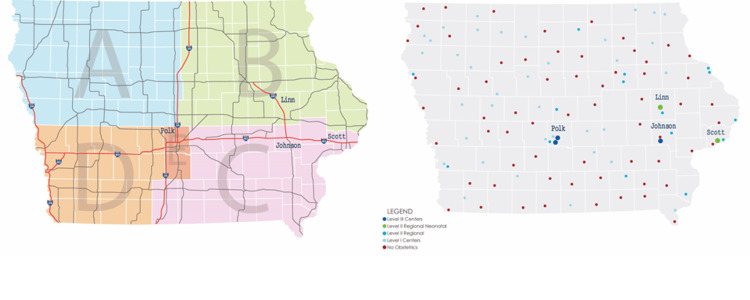
Maps of the State of Iowa Shown with Iowa Hospital Association Districts and Hospitals The left pane shows the five districts for hospital care [13] and explains our geographic modeling for care at the two private level III maternal care hospitals in Polk County in Central Iowa and the one university level III hospital in Johnson County in Southeast Iowa. The two Western districts A and D are combined because the patients would literally drive through Polk County on their way to Johnson County, two more hours from their residence. The left pane also shows the interstate highways directly connecting the counties. The right pane shows hospitals’ levels of maternal care, with obstetric and pediatric anesthesiologists required at the level III maternal care hospitals and the level II regional neonatology hospitals [6]. There were level II regional neonatal hospitals, also with obstetric anesthesiologists [6,7], in Linn and Scott Counties. From the left pane, these were located within 80-minute drives along two interstate highways to the university level III maternal care hospital in Johnson County. The post hoc analyses in the Results consider the effects of the two regional neonatology hospitals relatively close to Johnson County. Throughout the right pane, the many red dots show the scores of hospitals that have ceased obstetric care. The hospitals’ maternal care levels were current as of April 30, 2021, matching the studied data [7].

The state of Iowa has a land area larger than the state of New York but a population one-third that of New York City. Iowa functionally is divided into five hospital districts by interstate highways (Figure 1) [13]. There are three hospitals in the state that offer Level III maternal care (Figure 1) [7]. In Eastern Iowa, there is one such hospital, the University of Iowa, located in Johnson County, which has an obstetrics residency, an anesthesiology residency, and a nurse anesthetist training program. In the geographical center of the state (e.g., serving Western Iowa), in Polk County, the largest by population, there are two hospitals offering level III maternal care, neither with an obstetric nor anesthesia training program. Nurse anesthetist students do not rotate at those hospitals in Polk County.

Suppose that the distribution of women undergoing cesarean delivery and from counties without labor and delivery care were approximately equally distributed geographically between Western and Central Iowa (served by the two private level III hospitals, located in one county) and Eastern Iowa (served by the one university level III hospital). Then, patients from the rural counties may cause relatively equal distribution in cesarean deliveries and obstetric anesthesia workload at the two counties with level III maternal care (i.e., our hypothesis #2). In other words, the absence of local hospital labor and delivery care would prompt patients to the nearest level III hospital.

Dataset

The data studied included all discharges from hospitals in Iowa from October 1, 2015, through June 30, 2021, with an assigned major diagnostic category of pregnancy and childbirth, code 14 [5,14,15]. These administrative data were obtained from the Iowa Hospital Association. The start date corresponded to the state’s requirement to use the International Classification of Diseases, Tenth Revision, Procedure Coding System (ICD‑10‑PCS) [5,14]. The end date corresponded to the last date that the Iowa Hospital Association had released at the time of analysis. Among the 218,481 discharges, there were 60,905 with cesarean delivery [1]. Some patients having cesarean delivery near the end of June 2021 may not have been included in the dataset because they had not been discharged by June 30. Among patients with a delivery date of June 14, 2021, the study included all patients with a length of stay <17 postoperative days. Thus, including patients discharged through June 14, 2021, (2084 days) captured nearly all patients undergoing cesarean delivery. Consequently, we excluded the 371 patients with cesarean delivery occurring on June 15, 2021, or later. The remaining 60,534 cesarean deliveries were used in the statistical models (Table 1). For all patients, the county of residence was provided, which, in the rural state of Iowa with 99 counties (Figure 1), supplies sufficiently accurate, but deidentified, locations for distance assessments (i.e., analogous to using postal codes for modeling Houston).

**Table 1:** Characteristics of the Patients Undergoing Cesarean Delivery in the State of Iowa ^a^  All values in braces are % of the column with the denominator in the first row. All values in parentheses are row % (e.g.., in next row of #'s, 80.1% + 0.3% + 19.6% = 100%). In the third row from the bottom, the sum of the two-row column percentages equals 46.3%, meaning that fewer than half of the patients with comorbidities received care at the level III hospitals. There is no test because the column sizes are that of the population, not a sample. ^b^  Corresponding International Classification of Diseases, 10th revision, codes are given in the STATA code (StataCorp, College Station, TX) at: https://FDshort.com/CesareanWhatHospital. ^c^  For the counts from 1 to 9, we have labeled them as “<10” for potential confidentiality concerns.

Patient characteristics at each of the three groups of hospitals	73 hospitals with levels I or II maternal care	One university hospital with level III maternal care	Two private hospitals in the same county and with level III maternal care
Total # of cases	41,913	4451	14,170
Cesarean deliveries (% of row total = 60,534)	(69.2%)	(7.4%)	(23.4%)
Iowa Hospital Association Districts (Figure 1) {% of column total}^a^			
A, D, or E, Northwest, Southwest, or Central, excluding Polk County	12,603 (80.1%) {30.1%}	43 (0.3%) {1.0%}	3086 (19.6%) {21.8%}
Polk County	2291 (19.1%) {5.5%}	73 (0.6%) {1.6%}	9657 (80.3%) {68.2%}
B, Northeast Iowa	15,064 (91.6%) {35.9%}	1138 (6.9%) {25.6%}	246 (1.5%) {1.7%}
C, Southwest Iowa, excluding Johnson County	8511 (76.2%) {20.3%}	1517 (13.6%) {34.1%}	1149 (10.3%) {8.1%}
Johnson County	668 (30.4%) {1.6%}	1530 (69.6%) {34.4%}	<10
Not Iowa resident	2756 (93.8%) {6.6%}	150 (5.1%) {3.4%}	31 (1.1%) {0.2%}
Admitted ≥2 days before surgery or transferred from another hospital	2356 (47.8%) {5.6%}	1202 (24.4%) {27.0%}	1369 (27.8%) {9.7%}
Patient age ≥40 years	1081 (60.9%) {2.6%}	212 (11.9%) {4.8%}	483 (27.2%) {3.4%}
Severe preeclampsia or HELPP syndrome,^b^ principal diagnosis present on admission	905 (51.3%) {2.2%}	270 (15.3%) {6.1%}	588 (33.4%) {4.2%}
Placenta accreta, increta, or percreta,^b^ principal diagnosis	<10^c^	38 (70.4%) {0.8%}	<10^c^
Placenta previa,^b^ principal diagnosis	299 (47.2%) {0.7%}	111 (17.5%) {2.5%}	223 (35.2%) {1.6%}
Abruptio placentae,^b^ principal diagnosis	280 (65.1%) {0.7%}	44 (10.2%) {1.0%}	106 (24.6%) {0.8%}
Peripartum cardiomyopathy or diseases of the circulatory system,^b^ principal diagnosis	16 (30.2%) {0.0%}	18 (34.0%) {0.4%}	19 (35.8%) {0.1%}
Viral diseases complicating pregnancy,^b^ principal diagnosis, present on admission	<10^c^	<10^c^	<10^c^
One or more of the preceding 8 conditions	4611 (53.7%) {11.0%}	1522^a^ (17.7%) {34.2%}	2447^a^ (28.5%) {17.3%}
Commercial insurance	25,495 (68.3%) {60.8%}	2687 (7.2%) {60.4%}	9148 (24.5%) {64.6%}
Total hospital days, counted as midnight census, among the patients	125,561 (63.4%)	21,695 (11.0%)	50,736 (25.6%)

Statistical modeling

The results are written to be self-contained so that a reader less interested in the statistical details can skip to that section. For readers wanting more details or to replicate our work with data from a different state or province, the Stata version 17.0 (StataCorp, College Station, Texas) code and output in the sequence of these methods, the results, and tables are available at: https://FDshort.com/CesareanWhatHospital. Importantly, what makes our study unique is the focus of analyses on the hospitals and their total counts of cesarean deliveries [5,8,9], not why individual patients are having cesarean birth (see Discussion) [2,3].

Two binary dependent variables were created from the three columns of Tables 1-3. Specifically, the first binary variable was set equal to 1 if the patient underwent cesarean delivery at the university level III maternal care hospital in Johnson County (Southeast Iowa), and 0, otherwise (Figure 1). The second binary variable was 1 if the patient underwent cesarean delivery at one of the two private level III hospitals in Polk County (Central Iowa), and 0, otherwise (Figure 1). These are the three largest hospitals in Iowa by the number of hospital beds. Table 2 gives the distributions of hospitals by their Rural-Urban Continuum Codes and by their Iowa Hospital Association district [16].

**Table 2:** Characteristics of the 60,534 Cesarean Deliveries Among the Three Hospital Groups ^a^ This table gives the 2013 Rural-Urban Continuum Codes of the hospitals [16]. The nine codes of the patients are given in the STATA code (StataCorp, College Station, TX) at: https://FDshort.com/CesareanWhatHospital. All values in parentheses are row %, and those in braces are % of the column, with denominators in the first row.

Hospital characteristic among each of the three groups of hospitals	73 hospitals with levels I or II maternal care (61 Counties)	One university hospital with level III maternal care (Johnson County)	Two private hospitals with level III maternal care (Polk County)
Total # of cases	41,913	4451	14,170
Cesarean deliveries (% of row total = 60,534)	(69.2%)	(7.4%)	(23.4%)
Rural Urban Continuum Codes {% of column total}^a^			
2, County in metropolitan area of 250,000 to one million population	14,284 {34.1%}	-	14,170 {100%}
3, County in metropolitan area of fewer than 250,000 population	12,219 {29.2%}	4451 {100%}	-
4-5, Urban population of 20,000 or more	6330 {15.1%}	-	-
6-7, Urban population of 2500 to 19,999	8590 {20.5%}	-	-
8-9 Rural or less than 2500 urban population	490 {1.2%}		
Iowa Hospital Association Districts (% of row total) {% of column total}			
A, Northwest Iowa	11,097 (100%) {26.5%}	-	-
B, Northeast Iowa	15,215 (100%) {36.3%}	-	-
C, Southeast Iowa, including Johnson County	10,312 (69.8%) {24.6%}	4451 (39.2%) {100%}	-
D, Southwest Iowa	2203 (100%) {5.3%}	-	-
E, Central Iowa, Polk County and a rural adjacent county	3086 (17.9%) {7.4%}	-	14,170 (82.1%) {100%}

**Table 3:** Characteristics of the Patients’ Residence counties Among the Three Hospital Groups ^a^ The sum of cesarean deliveries in the second row equals 10,612. All values in parentheses are row %, and those in braces are % of the column, with denominators in the first row. ^b^ To interpret “consecutive quarters,” the 1st consecutive quarter was October 1, 2015, through December 31, 2015, 2nd was January 1, 2016, through March 31, 2016, …, 22nd was January 1, 2021, through March 31, 2021, and 23rd (incomplete) was from April 1, 2021, through June 14, 2021. Thus, for a patient with cesarean delivery on March 1, 2016, counts were made of the patient’s county during the period January 1, 2016, through March 31, 2016. ^c^ The counts of counties differ among consecutive quarters. The counts reported are the number of distinct counties in Iowa meeting the criterion for at least one of the studied quarters. ^d^ All discharges in the Iowa Hospital Association data include the National Provider Identifier of the physician performing the principal surgical procedure. The 60,534 NPI could be matched without any missing values to the physician’s self-reported principal specialty using the National Provider Identifier database.

Characteristics of the county of the patient’s residence	73 hospitals with levels I or II maternal care	One university hospital with level III maternal care	Two private hospitals in the same county and with level III maternal care
Total # of cases	14,913	4451	14,170
Cesarean deliveries (% of row total = 60,534)	(69.2%)	(7.4%)	(23.4%)
No cesarean delivery performed by any physician in the patient’s Iowa county with discharge during the concurrent quarter of patient’s delivery,^b^ 57^c^ of the 99 counties in Iowa (% of row total) {% of column total}	6970 ^a ^(65.7%) {16.6%}	783 ^a ^(7.4%) {17.6%}	2859 ^a ^(26.9%) {20.2%}
No cesarean delivery performed by an obstetrician^d^ in the patient’s Iowa county with discharge during the concurrent quarter of patient’s delivery,^b^ 77^c^ of the 99 counties in Iowa	11,982 (74.5%) {28.6%}	880 (5.5%) {19.8%}	3224 (20.0%) {22.8%}
No deliveries at the hospital in the patient’s Iowa county with discharge during the concurrent quarter of patient’s delivery,^b^ 54^c^ of the 99 counties in Iowa	6734 (65.6%) {16.1%}	748 (7.3%) {16.8%}	2776 (27.1%) {19.6%}
No cesarean delivery was performed by an obstetrician in the patient’s Iowa county or in a contiguous Iowa county, and with discharge during the concurrent quarter of patient’s delivery,^b^ 12^c^ of the 99 counties in Iowa, and four counties in last full quarter, all counties coincident to a state border	1044 (99.7%) {2.5%}	<10	40 (3.7%) {0.3%}
Cesarean deliveries (% of row total = 60,534)	(69.2%)	(7.4%)	(23.4%)
No cesarean delivery performed by any physician in the patient’s Iowa county with discharge during the concurrent quarter of patient’s delivery,^b^ 57^c^ of the 99 counties in Iowa (% of row total) {% of column total}	6970 ^a ^(65.7%) {16.6%}	783 ^a ^(7.4%) {17.6%}	2859 ^a ^(26.9%) {20.2%}

Several characteristics of patients’ counties are given in Table 3. The characteristic used for the modeling is from the second row: whether the patient’s residence county in Iowa had no cesarean deliveries during the quarter. By this, we mean that the data were segmented into consecutive quarters (e.g., the 1st quarter was October 1, 2015, through December 31, 2015, and the 20th quarter was July 1, 2020, through September 30, 2020). If the patient delivered on September 1, 2020, then the value of the variable was the count of cesarean deliveries in the patient’s county of residence between July 1, 2020, and September 30, 2020.

Hypothesis #1 was tested using the two binary dependent variables and various patient characteristics (Table 1 and Table 3). For each hospital, the coefficients of the Poisson regressions were calculated with robust variance estimation. There were separate regressions for each of the binary dependent variables because the estimates for the covariates differ significantly, reflecting different geographic locations of the hospitals and statewide planning (e.g., for adherent placentation) (Table 1). Poisson regression was used so that we could model the incremental risk ratios for the independent binary variables associated with the binary dependent variables (Table 4) [17]. The incremental risk ratios equal the exponentials of the estimated coefficients [17]. Logistic regression, in contrast, models odds ratios, not relative risks. The odds ratio is an unacceptably biased estimator for the relative risk when the dependent variable exceeds a 10% occurrence, as for the two private hospitals (Table 1) [18]. Examples in the Stata code at https://FDshort.com/CesareanWhatHospital show that odds ratios were misleading for quantitative comparisons between models, as was used to assess their face validity.

**Table 4:** Incremental Risk Ratios and 99% Two-Sided ConfidenceIntervals Abbreviations: IRR, incremental risk ratio; CI, confidence interval; LCL, lower confidence limit; UCL, upper confidence limit ^a^ The university hospital had multiple anesthesia training programs, including anesthesiology residency, fellowships, and the state’s sole nurse anesthesia training program. The two private hospitals had neither an anesthesia nor an obstetrics training program. ^b^ The STATA code  (StataCorp, College Station, TX) at https://FDshort.com/CesareanWhatHospital shows similar incremental risk ratios when instead the factor is either (a) no cesarean delivery by an obstetrician in the patient’s county during the quarter of the patient’s delivery or (b) no Major Diagnostic Category 14, pregnancy and childbirth, in the patient’s county during the quarter of delivery. ^c^ Hypothesis #1 was that both hospitals would have incremental risk ratios >1.0 among patients with no cesarean delivery during the quarter of their surgery (Table 3). Therefore, the P‑values for these two rows are reported with Bonferroni adjustment. The other rows are covariates, the estimates relevant to the extent that they show face validity to the model (e.g., patients with adherent placenta receive care at the university hospital).

Level III maternal care group	Binary variable in Tables 2 and 3	IRR	99% LCL	99% UCL	P-value
University hospital^a^	Cesarean delivery, none in patient’s county during the quarter^b^	1.61	1.46	1.78	<0.001^c^
	Residence Districts A, D, and E, Western Iowa	0.05	0.03	0.07	<0.001
	Residence Polk County, location level III hospitals	0.12	0.08	0.17	<0.001
	Residence District B, Northeast Iowa	1.20	0.97	1.48	0.029
	Residence District C, not Johnson County	2.27	1.85	2.80	<0.001
	Residence Johnson County, a level III hospital	12.47	10.17	15.29	<0.001
	Preoperative admission, transfer	3.02	2.79	3.26	<0.001
	Patient age ≥40 years	1.21	1.04	1.40	0.001
	Preeclampsia	1.37	1.18	1.58	<0.001
	Adherent placenta	4.99	2.78	8.94	<0.001
	Placenta previa	1.79	1.43	2.24	<0.001
	Abruptio placentae	1.13	0.82	1.56	0.31
	Cardiomyopathy, CV disease	3.81	2.32	6.28	<0.001
	Commercial insurance	0.89	0.83	0.95	<0.001
Two private hospitals^a^	Cesarean delivery, none in patient’s county during quarter	4.51	4.20	4.84	<0.001 ^c^
	Residence Districts A, D, and E, Western Iowa	8.74	5.49	13.90	<0.001
	Residence Polk County, location level III hospitals	76.47	48.28	121.12	<0.001
	Residence District B, Northeast Iowa	0.84	0.51	1.37	<0.001
	Residence District C, not Johnson County	5.73	3.58	9.15	<0.001
	Residence Johnson County, a level III hospital	0.04	0.00	0.58	0.002
	Preoperative admission, transfer	1.24	1.19	1.30	<0.001
	Patient age ≥40 years	1.06	0.99	1.14	0.033
	Preeclampsia	1.37	1.29	1.47	<0.001
	Adherent placenta	0.42	0.17	1.04	0.013
	Placenta previa	1.28	1.16	1.42	<0.001
	Abruptio placentae	1.19	1.02	1.40	0.004
	Cardiomyopathy, CV disease	1.19	0.86	1.64	0.17
	Commercial insurance	1.08	1.05	1.11	<0.001

Hypothesis #1 was a test of the significance of one binary variable only: whether the patient’s residence county had no cesarean deliveries during the quarter (Table 3). The estimated coefficients of the other variables in the model were checked for face validity (e.g., comparison between models), but were treated only as covariates (Table 4). Because our hypothesis #1 was a test for a binary variable from two regressions, the two P‑values were reported with Bonferroni adjustment for the two comparisons (Table 4). Because our study size was based on dates and included every cesarean delivery but cannot be increased, to be conservative, we treated P < 0.01 as statistically significant and report 99% confidence intervals. If 10% of the healthy patients from counties with no cesarean deliveries received care at a level III hospital, and that was increased to 14%, the Cohen’s D effect size would be small [19]. Therefore, we considered a lower confidence limit of 1.40 or larger to be small but important.

Hypothesis #2 was tested by calculating the predicted probabilities, first with all data as observed, and second while changing one factor at a time to represent potential modifications through policy changes (Table 4) [20]. The resulting means of the probabilities are similar to adjusted treatment means of the analysis of variance, but for our nonlinear regression models. The means of the probabilities were estimated using logistic regression. Robust variance estimation was used for potential model misspecification (e.g., from unmeasured covariates influencing patient decision-making and the assumptions of linearity).

In the Stata code at https://FDshort.com/CesareanWhatHospital, we include an example showing that the means of the probabilities can sum to a value greater than one for each factor when calculated using Poisson regression. The parameter estimates from the logistic regression models matching the Poisson regression models of Table 4 are given in the Stata code at https://FDshort.com/CesareanWhatHospital.

For testing hypothesis #2, the variables considered potentially modifiable were those in the model (Table 4) that were unrelated to patients’ residences or hospitals’ counties. Those variables are the ones listed in Table 5. For each of the potentially modifiable variables, we calculated the difference of the predictive margin with the binary factor being absent for all cesarean deliveries (e.g., no patient has adherent placentation) versus the predictive margin using the observed grand mean of the variable (Table 5). All other factors were kept unchanged. That was done because many factors were binary variables with no intersection (e.g., no patient from Johnson County where the university hospital was located and in southeast Iowa was from the northwest district of the State) (Table 1). To report these differences in units of cases per week, the differences in mean probabilities were then multiplied by 200 cesarean deliveries per week, where 200 was the average over the study period. The 99% confidence intervals for differences in cesarean deliveries were calculated using the delta method. We treated changes of at least one case per week based on the confidence intervals to be managerially relevant (Table 5).

**Table 5:** Effect of Removing Types of Patients, Programs, and Features on Weekly Cesarean Deliveries at the Two Hospital Groups with Level III Maternal Care ^a^ The statistical models are those given in Table 4. The university hospital had multiple anesthesia training programs, including anesthesiology residency, fellowships, and the state’s sole nurse anesthesia training program. The two private hospitals had neither an anesthesia nor an obstetrics training program. ^b^ The STATA code (StataCorp, College Station, TX) at https://FDshort.com/CesareanWhatHospital shows incremental risk ratios when instead the factor is no cesarean delivery by an obstetrician in the patient’s county during the quarter of the patient’s delivery. Point estimates are the same. ^c^ For example of changes, patients currently transferred to the university hospital or admitted days before delivery would not receive care at the hospital. For example, all patients could undergo cesarean delivery in their county of residence. Finally, all patients would have Medicaid insurance. These examples, each statistically significant, include some changes that are negative valued, showing decreases and some positive valued, showing increases. ^d^ Change based on the 99% confidence interval exceeding one case per week.

Level III maternal care hospital(s)	Binary variable = 1 (Yes) in Tables 2 to 4^c^	Change in cases per week if value of binary variable in preceding column = 0^c^ (99% confidence interval)
University hospital^a^	Cesarean delivery, none in patient’s county during quarter^b^	-1.09 (-1.3, -0.8)
	Preoperative admission, transfer	-2.2 (-2.4, -2.0)^c,d^
	Patient age ≥40 years	-0.1 (-0.2, 0.2)
	Preeclampsia	-0.2 (-0.2, -0.1)
	Adherent placenta	-0.1 (-0.1, -0.1)
	Placenta previa	-0.1 (-0.2, -0.1)
	Abruptio placentae	0.0 (0.0, 0.0)
	Cardiomyopathy, cardiovascular disease	0.0 (-0.1, 0.0)
	Commercial insurance	1.0 (0.4, 1.6)
Two private hospitals^a^	Cesarean delivery, none in patient’s county during quarter	-5.81 (-6.1, -5.5)^c,d^
	Preoperative admission, transfer	-0.9 (-1.1, -0.7)
	Patient age ≥40 years	-0.1 (-0.2, 0.0)
	Preeclampsia	-0.5 (-0.7, -0.4)
	Adherent placenta	0.0 (0.0, 0.0)
	Placenta previa	-0.2 (-0.2, -0.1)
	Abruptio placentae	-0.1 (-0.1, 0.0)
	Cardiomyopathy, cardiovascular disease	0.0 (0.0, 0.0)
	Commercial insurance	-2.4 (-3.2, -1.6)^c,d^

## Results

Description of the study population

Among the 60,534 cesarean deliveries analyzed, 7.4% were performed at the university level III hospital (Tables 1-2). Although local physicians may have been familiar with the university hospital, there were 23.4% of the cesarean deliveries performed at private hospitals with level III maternal care but no obstetric or anesthesia training programs (Tables 1-2). The level III hospitals cared for fewer than half the patients with obstetric risk factors who had a cesarean delivery (46.3%; Table 1).

There were 10,612 cesarean deliveries among patients residing in an Iowa county with no cesarean births in their county during the quarter of the patient’s delivery (Table 3). There were 7.4% (783) performed at the level III university hospital. There were 26.9% (2859) performed at the two other level III hospitals, 3.6-fold (2859/783) more than at the university hospital.

The 10,612 cesarean deliveries were for patients who resided in 57 distinct counties, among the 99 in Iowa. Most of these cesarean deliveries were among patients living in counties with a hospital (77%, 8202/10,612), but not with a hospital providing labor and delivery care.

The two private hospitals were part of the UnityPoint Health and Mercy Medical networks of hospitals. Although we do not know what referral networks and other arrangements may have been present, few cesarean deliveries (8%, 872) were among patients living in counties with a hospital in a network encompassing the private level III hospitals (i.e., such relationships could not substantively influence results).

Tests of the two hypotheses

Patients from counties without cesarean births during the quarter of their surgery (Table 3) disproportionately received care at the level III maternal care hospitals, rather than at levels I and II hospitals (Table 4). The lower 99% confidence limits for the incremental risk ratios were 1.46 and 4.20, respectively. For example, a patient from a county without a hospital labor and delivery unit during the quarter had approximately a 46% greater probability of undergoing cesarean delivery at the level III maternal care hospital in Johnson County versus a patient with otherwise matching covariates and from a county with a level I or II hospital. Because both incremental risk ratios exceeded 1, hypothesis #1 was supported.

The cesarean deliveries among patients without hospital labor and delivery care in their counties were distributed unequally among the counties of the hospitals with level III maternal care. There were approximately 5.81 (standard error 0.11) extra cesarean delivery per week at the private hospitals versus 1.09 (standard error 0.10) at the university hospital (Table 5). Therefore, our hypothesis #2 was not supported. The 5.4-fold (5.81/ 1.09) relative effect between the two groups of hospitals shows the disparate influence of the absence of rural obstetrics programs on cesarean deliveries at level III maternal care hospitals.

Post hoc observations

We repeated the Poisson regression weighted by patients’ hospital lengths of stay in days. The incremental risk ratio was 1.32 (99% confidence limit 1.16 to 1.50) for the level III maternal care hospital in Johnson County. The ratio was 4.01 (3.64 to 4.42) for the level III hospitals in Polk County. Therefore, hypothesis #1 was supported, when considered in terms of labor and delivery ward census.

We examined some causes of the disparate (5.4-fold) effect on the level III hospitals of cases among patients without a local hospital for cesarean delivery. As above, neither training programs nor corporate referral networks can explain the result. Geography alone also cannot explain the finding because more than half (52%; 5502/10,612) of the patients undergoing cesarean delivery and residing in a county without hospital labor and delivery wards in that county during the quarter were from the eastern districts B and C, closer to the university hospital (Figure 1). (Note that there are no confidence intervals or P-values because every cesarean delivery in the state was included, not a sample.) There was an effect of the patient’s insurance, with substantively more patients with commercial insurance receiving care at the private level III hospitals (Table 5). In addition, the competitive effect of other hospitals with similar services had an effect (Figure 1). Specifically, the eastern districts included the state’s two level II regional neonatal centers (see Introduction for definitions). Those hospitals performed 801 of the 5502 cesarean deliveries among the patients from a county with no such care, similar to the 783 such cases at the level III university hospital.

## Discussion

We studied patients who must leave their home counties for hospital labor and delivery services because the hospitals in their counties do not offer such care. Our results for hypothesis #1 show that these patients disproportionately (i.e., incremental risk ratio >1.0) go to level III hospitals (e.g., with obstetric anesthesiologists). In other words, the patients are not receiving care at the level III hospitals because the patients are high-risk. Reemphasizing for clarity, although patients at high-risk do, of course, tend to go to high-acuity hospitals [21], our novel results show that these risk factors account for few of the extra patients at the level III hospitals. Rather, the hospitals are large or the specialty care has a marketing or referral attraction. However, from our results for hypothesis #2, the distribution of these extra cases among counties was unequal, far more than expected based on geography. Both patients’ insurance and the availability of large level II hospitals with many cesarean deliveries also influenced caseloads. These results show and explain why level III hospitals would best forecast their longitudinal changes in cesarean deliveries and obstetrical anesthesia workload using time series analyses of their own data [8]. It is much simpler for a hospital to analyze its own operating room, anesthesia health record, or anesthesia billing data rather than incorporating state or provincial data into these calculations. Knowing that there would be unlikely incremental value for forecasting in using other hospitals’ data is reassuring.

Considering further our findings for hypothesis #1, we are unaware of a way to infer why many patients from counties without hospital labor and delivery care went preferentially to the level III maternal care hospitals, even though for most patients there are many closer levels I and level II hospitals, all performing cesarean delivery [3,6] (Figure 1). Perhaps that makes our results more important because they are not directly predictable from earlier studies of patients’ preferences [22,23]. For example, although most gravid women report that quality of care is important in choosing a hospital for obstetrics, most did not know what quality metrics to consider and gave a low priority to ones reported publicly [22]. Preference for choosing the level III hospitals may be caused simply by those hospitals being large, thus performing the most cesarean deliveries among all Iowa hospitals during the study period. Size is a factor related to market visibility, from the hospital being heard about more [9,10]. A previous national survey of United States women found that those living in non-metropolitan areas were more concerned about overall reputation than the logistics of location and insurance when choosing a hospital for obstetric care [23]. Level III hospitals may have a stronger reputation for quality either because they meet level III standards or because reputation is based on having many obstetric patients (i.e., the hospital name is known).

Considering further our findings for hypothesis #2, controlling for geography and maternal risk factors, the patients with commercial insurance were more likely to undergo cesarean delivery at one of the private level III hospitals than the university hospital (Table 4-5). The type of insurance was a covariate, not a factor related to our hypotheses. We do not know how that result can be used in a generalizable manner by hospitals other than those we studied. Also, we do not recommend generalizing the observation beyond cesarean delivery. An earlier study using Iowa data showed that there was a statistically significant but quantitatively unimportant effect of type of insurance on the overall relative distribution of all types of major therapeutic procedures, including cesarean delivery, among hospitals in Iowa [24].

We have provided the methodological steps and Stata code that others can follow at https://FDshort.com/CesareanWhatHospital, including examples showing that these are in no way “obvious,” with logistic regression giving biased estimates for some questions (Table 4) and Poisson regression giving biased estimates for others (Table 5).

A limitation of our work is that there is a lack of other studies to which comparison can be made to judge the generalizability of our findings to other rural states and provinces outside the United States. That is a consequence of our work being novel in examining the impact of patients from counties without hospital labor and delivery units on cesarean deliveries at level III maternal care hospitals. However, the context of the problem with rural hospitals closing obstetric programs is occurring throughout the United States, not only in Iowa [3]. For interested readers, the different question of factors predicting when women from rural areas do not receive care at a local hospital for childbirth (i.e., not limited like our study to patients with no local hospital and not limited to cesarean births), we recommend the 2016 study co-authored by Kozhimannil [21].

## Conclusions

Rural hospitals are shutting down their obstetric programs. This is because of an inadequate obstetrical workforce, not an insufficient number of anesthesia practitioners. Our results show that patients from counties without a hospital labor and delivery ward preferentially (and substantively) receive care at level III maternal care hospitals, bypassing many hospitals with levels I and II maternal care. The type of insurance and the competitive effect of large level II hospitals with similar services are important considerations. For predicting cesarean deliveries at level III hospitals with obstetric anesthesiologists, geography alone is an insufficient predictive factor for patient choice.

## References

[1] Handley SC, Passarella M, Srinivas SK, Lorch SA (2021). Identifying individual hospital levels of maternal care using administrative data. BMC Health Serv Res.

[2] Hung P, Henning-Smith CE, Casey MM, Kozhimannil KB (2017). Access to obstetric services in rural counties still declining, with 9 percent losing services, 2004-14. Health Aff (Millwood).

[3] Carrel M, Keino BC, Ryckman KK, Radke S (2022). Labor & delivery unit closures most impact travel times to birth locations for micropolitan residents in Iowa. J Rural Health.

[4] (2022). Iowa Department of Public Health. Division of Health Promotion & Chronic Disease Prevention -Bureau of Family Health. Access to Obstetrical Care in Iowa: A Report to the Iowa State Legislature - Calendar Year 2019. Calendar Year.

[5] Thenuwara K, Dexter F, Radke S, Epstein RH (2022). Cesarean delivery availability in Iowa was not constrained by anesthesia workforce limitations: retrospective cohort study of inpatient surgery case counts. Periop Care Oper Room Manag.

[6] Iowa Administrative Code (2022). Iowa Administrative Code. Chapter 150, Iowa regionalized system of perinatal health care. Chapter.

[7] (2022). Iowa Department of Public Health. Iowa Hospitals with Obstetric Delivery Services (updated 4-30-2021). https://idph.iowa.gov/Portals/1/userfiles/88/IOWA%20HOSPITALS%20WITH%20OBSTETRIC%20DELIVERY%20SERVICES_4_30_21%20-%20with%20counties%20%281%29.pdf.

[8] Dexter F, Epstein RH, Thenuwara KN (2022). Long-term capacity planning for obstetric surgical suites using quantile linear regression (IN PRESS). Anaesth Intens Care.

[9] Dexter F, O'Neill L (2004). Data envelopment analysis to determine by how much hospitals can increase elective inpatient surgical workload for each specialty. Anesth Analg.

[10] Dexter F (2017). Factors substantively influencing numbers of surgical cases performed at a research hospital. Ann Res Hosp.

[11] Henning-Smith C, Almanza J, Kozhimannil KB (2017). The maternity care nurse workforce in rural U.S. hospitals. J Obstet Gynecol Neonatal Nurs.

[12] Dexter F, Wachtel RE, Sohn MW, Ledolter J, Dexter EU, Macario A (2005). Quantifying effect of a hospital's caseload for a surgical specialty on that of another hospital using multi-attribute market segments. Health Care Manag Sci.

[13] Iowa Hospital Association (2022). Iowa Hospital Association. https://www.ihaonline.org/iha-districts/.

[14] Iowa Hospital Association (2022). Data Dictionary For Use with the IHA Inpatient Database. https://www.ihaonline.org/wp-content/uploads/2020/12/IP-Data-Dictionary-2015-Q2.pdf.

[15] (2022). United States Senate Committee on Veterans’ Affairs. U.S. Department of Veterans Affairs Maternity Services: Actions Needed to Enhance Communication and Plan for Coordination of Care in the Community. https://www.veterans.senate.gov/imo/media/doc/FINAL%20Report-%20VA%20Maternity%20Services%20Dec%202016.pdf.

[16] (2022). U.S. Department of Agriculture, Economic Research Service, Rural-Urban Continuum Codes. https://www.ers.usda.gov/data-products/rural-urban-continuum-codes/.

[17] Chen W, Shi J, Qian L, Azen SP (2014). Comparison of robustness to outliers between robust poisson models and log-binomial models when estimating relative risks for common binary outcomes: a simulation study. BMC Med Res Methodol.

[18] Prasad K, Jaeschke R, Wyer P, Keitz S, Guyatt G (2008). Tips for teachers of evidence-based medicine: understanding odds ratios and their relationship to risk ratios. J Gen Intern Med.

[19] Chen H, Cohen P, Chen S (2010). How big is a big odds ratio? Interpreting the magnitudes of odds ratios in epidemiological studies. Commun Stat - Simul Comput.

[20] UCLA: Statistical Consulting Group (2022). UCLA: Statistical Consulting Group. Using margins for predicted probabilities. https://stats.oarc.ucla.edu/stata/dae/using-margins-for-predicted-probabilities.

[21] Kozhimannil KB, Casey MM, Hung P, Prasad S, Moscovice IS (2016). Location of childbirth for rural women: implications for maternal levels of care. Am J Obstet Gynecol.

[22] Gourevitch RA, Mehrotra A, Galvin G, Karp M, Plough A, Shah NT (2017). How do pregnant women use quality measures when choosing their obstetric provider?. Birth.

[23] Hebert LE, Freedman L, Stulberg DB (2020). Choosing a hospital for obstetric, gynecologic, or reproductive healthcare: what matters most to patients?. Am J Obstet Gynecol MFM.

[24] Dexter F, Jarvie C, Epstein RH (2018). Lack of a substantive effect of insurance and the national US payment system on the relative distribution of surgical cases among hospitals in the State of Iowa: a retrospective, observational, cohort study. J Clin Anesth.

